# Chronic radial head dislocation caused by a rare solitary osteochondroma of the proximal radius in a child: a case report and review of the literature

**DOI:** 10.1186/s13104-015-1095-y

**Published:** 2015-04-08

**Authors:** Xiao-feng Niu, Jian-hua Yi, Jun Hu, Liang-bao Xiao

**Affiliations:** Department of Upper Extremity Orthopedics, Eastern Hospital of the First Affiliated Hospital, Sun Yat-sen University, Guang Zhou, China

**Keywords:** Solitary osteochondroma, Radial head, Dislocation, Osteotomy

## Abstract

**Background:**

Osteochondroma is the most common benign bone tumor of the upper limbs that occurs during the developmental phase of children. Solitary epiphyseal enchondromas can be usually found in the humeral capitellum, and the proximal ulna of the elbow.

**Case presentation:**

Herein, we report the case of a 12-year-old boy of Han ethnicity with a developmental radial head dislocation with a progressive radius deformities, caused by a solitary osteochondroma which originated from the proximal metaphysis of the radius. Obvious complaints and limitations were present. After tumor excision was performed, radial head reduction and deformity correction were achieved through a biplanar shortening osteotomy of the radius.

**Conclusions:**

After a follow-up of 18 months, the child remained asymptomatic and regained a full range of motion. Radiographic study revealed satisfactory reduction of the radial head with no recurrence of the osteochondroma.

## Background

Radial head dislocations occurring in children or adolescents can be caused by non-traumatic lesions including congenital malformations, infections, and tumors in bones and joints of the forearms. Osteochondroma is the most common benign bone tumor in perichondral bones, and it may occur in the proximal metaphysis of a long bone around the joints. The tumor is also known as an epiphyseal enchondroma, and can present as an exogenous mass deviating from the normal direction of osteogenesis in the epiphysis. Tumors occurring in the epiphyseal ends of radius and ulna during the growth period can invade the unclosed epiphyses, and result in asymmetrical and abnormal growth in both the radius and ulna. There are usually symptoms such as developmental malformations in the forearm bones, wrist and elbow joint dislocations, and limited mobility. A majority of tumors leading to a progressive dislocation of the radial head involve the distal ulna [1], and are mainly seen in hereditary multiple exostoses (HME). Local bone or cartilage lesions in the distal ulna can cause changes in the distal epiphysis and secondary ulna and radius dysplasia, and this process is a result of the combined action of biological and biomechanical factors [2]. There are various treatments available, but the efficacy of treatments is still very limited [3,4].

Solitary osteochondroma originating from the elbow (distal humerus, proximal radius and ulna) is a rare disease, and it can cause local symptoms and signs through the progressive invasion of the epiphysis. The goal of treatment is to remove the lesion, correct deformities, rebuild or restore the joint stability and forearm rotation function [4], and provide a good environment for their sustained development. Herein, we report the diagnosis and treatment of a solitary osteochondroma of the proximal radius and review the literature of this condition.

## Case presentation

**Figure 1:**
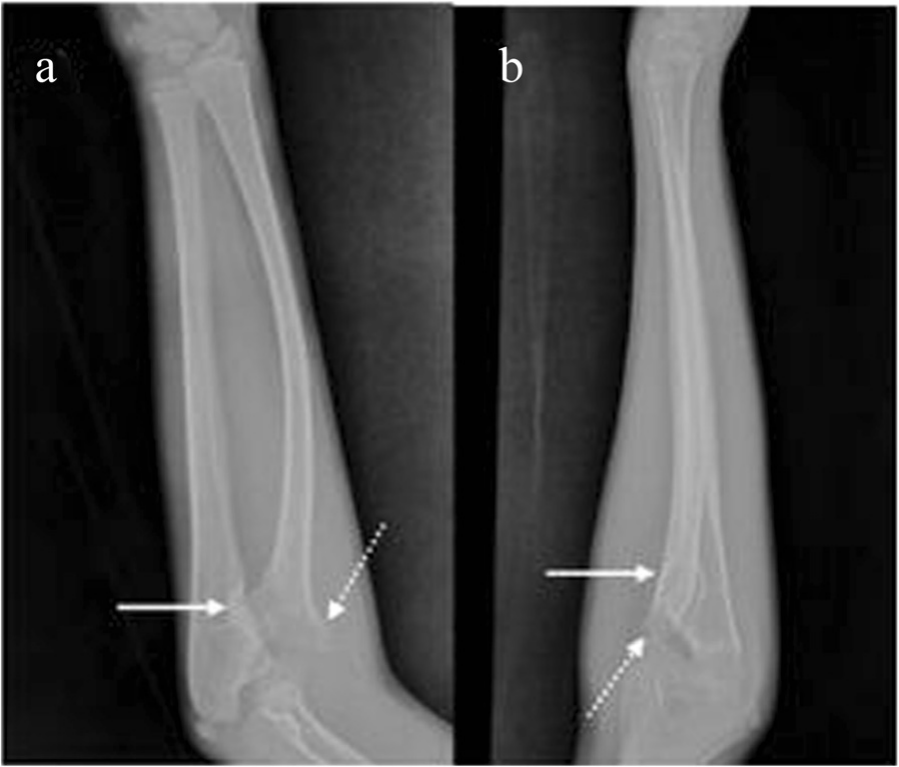
**Preoperative radiographs. (a)** Oblique and **(b)** anteroposterior radiographs of the right forearm showed a wide basal bony protuberance (solid arrow) in the dorsal metaphysis of the right proximal radius,and local depression with a dense edge;radial deformities in the proximal end, the radial shaft bending dorsally, ossification center shrinkage, and anterolateral dislocation (dotted arrow) were noted.

A 12-year-old male of Han ethnicity presented with a 1.5 year history of persistent pain, deformity, and limited mobility of his right elbow and forearm. He had undergone non-surgical treatment, which was ineffective and the symptoms progressively worsened. There were no other associated malformations or syndromes, no history of trauma, and no family history of bone cancer. Physical examination revealed the forepart of the right elbow was more raised compared with the left side, a bony mass was palpable, and there was no tenderness and no feeling of bounce or reduction when pressing down. The scope of activity of the right elbow joint and forearm was significantly limited (elbow 80° flexion, 20° extension, and forearm 10° pronation, 15° supination), and no injuries of the extremity nerves or blood vessels were found. Plain X-ray of the right forearm showed a wide basal bony protuberance in the dorsal metaphysis of the right proximal radius, radial deformities, a small radial head with anterolateral dislocation, the radial shaft bending towards to the dorsal ulnar side, and bone absorption in the corresponding ulna. The ulna had no significant shortening or angular, varus, or valgus deformities. X-ray examinations of other parts showed no tumor lesions (Figure 1). Based on the history and clinical findings, osteochondroma was considered.

**Figure 2:**
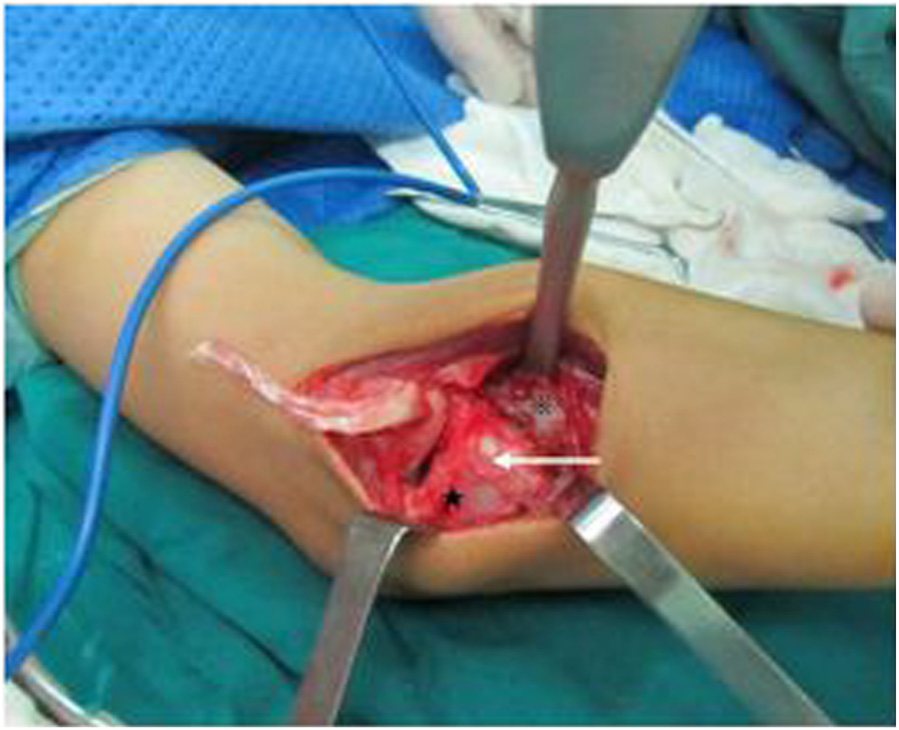
**Intraoperative image.** The tumor was located in the inner rear side of the radial neckand measured 2.8 × 2.6 × 2.5 cm, and outward expansion of the tumor caused a local depression in the adjacent ulna.

Surgical intervention was deemed necessary based on the tumor site, radial deformity, and limited mobility. However, the child was too young to perform resections of the radial head and segments invaded by tumor, which might lead to a secondary elbow valgus deformity and distal radioulnar joint dislocation. At surgery, the complete bone lesion and dislocated humeroradial joint were exposed through an elbow Kocher incision. The tumor was located in the inner rear of the radial neck. The upper edge of the tumor was about 0.5 cm from the articular surface of the radius. The base of the tumor was wide, and fused with the radius cortex without discontinuity. The outward growth of the tumor caused a local dent in the adjacent ulna. A clear dividing line between the tumor and the surrounding soft tissue was present, and there was no evidence of invasion (Figure 2). The radial tuberosity and the radial notch outside of the ulnar styloid process had been destroyed, and the radial head and metaphysis were significantly distorted with loss of the normal shallow disc-shaped concave surface. The humeroradial joint was filled with scar tissue, the residual annular ligament had obvious atrophy and degeneration, and the joint capsule was contracted.

**Figure 3:**
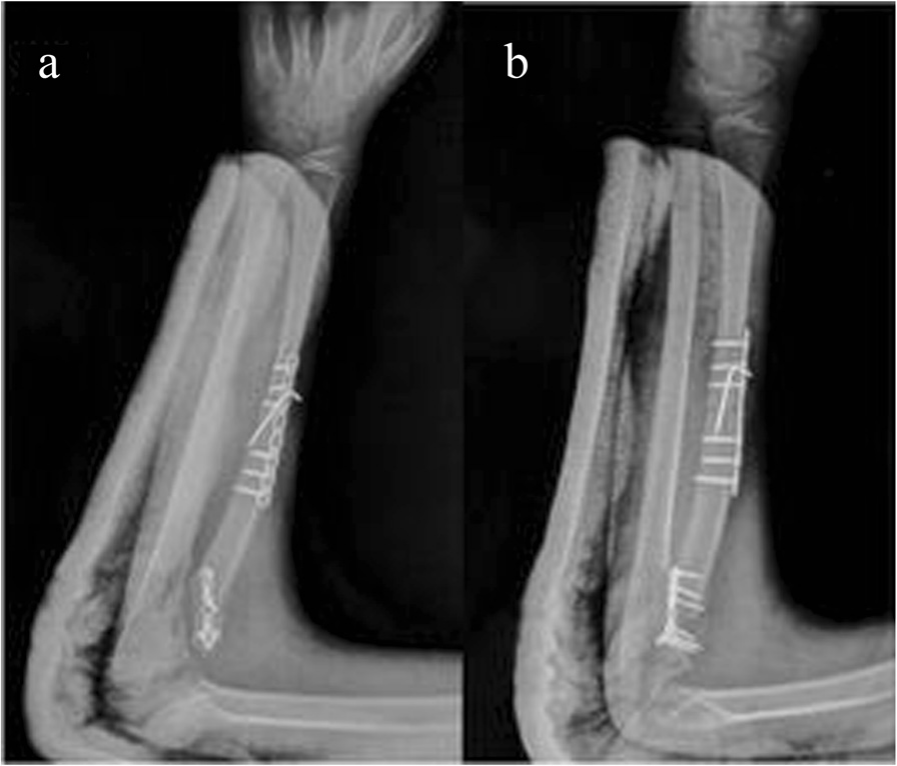
**Postoperative radiographs. (a)** Oblique and **(b)** lateral postoperative radiographs showing the resection position, the inner fixation, deformity correction, and restored anatomical relationship of the humeroradial joint.

While protecting the epiphyseal cartilage, complete marginal resection of the tumor and its capsule was performed. The cortical and cancellous bone of the tumor, and a little normal bone were chiseled to avoid leaving debris in the cartilage cap. Brain medical cotton and bone wax were used to reduce bleeding. The hyperplastic scar tissue within the articular cavity was thoroughly removed, and stripping of the soft tissue near the proximal radius was minimized to avoid damaging the blood supply of the radius and maintain its stability. The radial neck was cut off and fixed with a 2.0 mm T-type bone plate after the proximal end was rotated outward about 45°. A Henry incision in the middle of the forearm was made to expose the dorsal radius with the most obvious deformities. After the wedge osteotomy was completed (approximately 20°), a 2.5 mm straight bone plate and Kirschner wire were used for fixation. The radial head was reduced, and the anatomical relationship of the humeroradial joint was restored. A deep fascia pedicle from the right forearm was used to reconstruct the annular ligament. The osteotomy site was filled with attached tissues. Since there was no significant bending angular deformity that hindered reduction of the radial head, and the lengths of bilateral ulnas were similar, prolongation of the ulna was not necessary (Figure 3). The stability of the radial head and tightness of the annular ligament were assessed intraoperatively. Flexion and extension of the elbow and rotation of the forearm could be carried out passively. Free rotation of the radial head without further dislocation indicated a suitable tightness. Pathological examination of the resected specimen revealed osteochondroma, which was consistent with the preoperative diagnosis.

**Figure 4:**
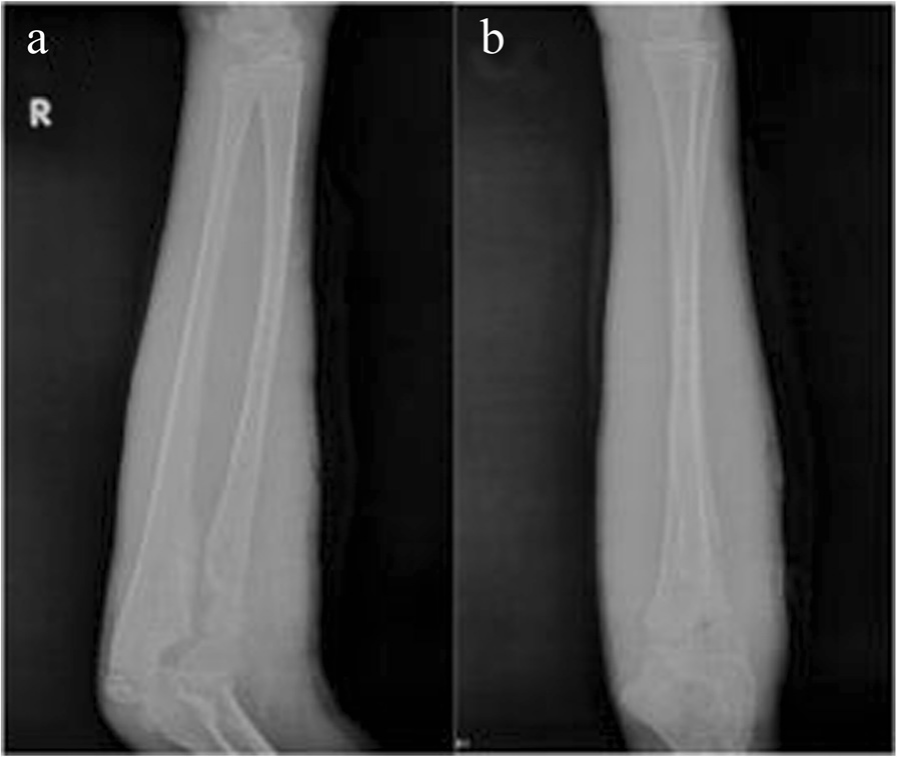
**Follow-up radiographs. (a)** Oblique and **(b)** anteroposterior radiographs at 16 months after surgery showed that the anatomical relationship of the humeroradial joint was normal; the osteotomy site was healed well without malunion or ischemic necrosis; and no tumor recurrence was present.

Postoperatively, the right upper limb was externally fixed in a plaster cast with 90° of elbow flexion and forearm supination. Active functional exercises were started 4 weeks after surgery. X-ray examination at 16 months showed no recurrence of humeroradial joint dislocation, the osteotomy site was healed well without malunion or ischemic necrosis, and no tumor recurrence. The internal fixators were removed by reoperation (Figure 4), and rehabilitation was started. Two months later, the patient regained normal function. The range of motion was full compared to the normal side.

## Conclusions

The most common pathogenic sites of osteochondroma are the distal femur and proximal tibia [5,6]. Although the exact pathogenic mechanism not been determined, it is believed related to cartilaginous ossification of cartilage tissue of the epiphyseal growth plate and epiphyseal dysplasia with separation [7]. Due to differences in locations and tumor size, the clinical manifestations of osteochondroma are diverse. Osteochondromas occurring between the ulna and radius in the elbow typically have no early symptoms, but as the tumor grows symptoms can include local pain, limited mobility (mainly forearm rotation), and skeletal deformities [1,2,8,9]. It has been reported that osteochondroma causing radial head dislocation is commonly seen in HME [10]. Masada *et al*. [3] classified the forearm deformity caused by HME into three groups, among which type IIa is radial head dislocation caused by an osteochondroma in the proximal radius that is accompanied by varying degrees of change in the “ulnar bow sign”. Solitary osteochondromas originating from the proximal radius are very rare [11,12], and it is difficult to determine the type.

With expansive growth of a lesion, complications may occur including pathological fractures, vascular and nerve damage, synovial cysts, infection, and malignant transformation [13]. The patient presented was treated late, and his symptoms were severe. If the disease course is long, adaptive pathological changes such as radial head deformation or a bow-shaped deformity caused by radial overgrowth may occur secondary to chronic joint dislocation [14]. These changes can make the radial longitudinal axis deviate from the capitellum ossification center, and therefore make it difficult to reduce the radial head. In this regard, restoration of the normal mechanical axis of the forearm through corrective osteotomy is required, which is conducive to reduction and maintenance of the radial head.

Osteotomy to lengthen the ulna and/or shortening the radius is supported by a number of studies [15,16]. An ideal osteotomy plane and angle can assure stability of the radial head, and partially improve forearm rotation [17]. Currently, there is no consensus on the necessity of repair of radial head dislocation with annular ligament damage and reconstruction methods [18-21]. If intraoperative exploration shows that the annular ligament has withered, degenerated, or even disappeared, and it cannot be repaired or restored, this is considered an unfavorable factor in maintaining radial head reduction. A variety of materials have been studied for annular ligament reconstruction including dorsal forearm fascia strips, autologous tendons, allogenic tendons, and silicone strips [14,22,23]. However, there are issues with most materials, and restricted forearm rotation is an unavoidable adverse outcome [24]. In this regard, some scholars have proposed that a deep fascia pedicle flap with autologous blood supply from the forearm should be transferred to reconstruct the annular ligament. This method reduces the possibility of fibrosis, and contributes to self-healing. The biomechanical performance after reconstruction is more ideal, and it is also conducive to stability of the radial head [25]. For our case, we chose a deep fascia pedicle flap as the graft material to reconstruct the annular ligament. The proximal radial osteotomy, radial head reduction, fascia incision, and annular ligament reconstruction were completed within the same incision, and the surgical procedure was relatively simple with less collateral damage to the blood vessels and nerves. Long-term observation revealed that development and function of the forearm extensor group were intact.

In summary, the choice of surgical approach should conform to the principle of individualization as much as possible to facilitate late functional recovery for young patients. Resecting the tumor and eliminating extrinsic compression can reduce secondary skeletal deformities and adaptive changes in the joint, providing a good environment for normal bone development. Radial osteotomy can balance the abnormal load of the elbow joint, and it is conducive to reduction and maintenance of the radial head.

## Consent

Written informed consent was obtained from the patient’s parents for publication of this Case Report and any accompanying images. A copy of the written consent is available for review by the Editor-in-Chief of this journal.

## Abbreviations

HME: Hereditary multiple exostoses

## References

[1] Arms DM, Streeeker WB, Manske PR (1997). Management of forearm deformity in multiple hereditary osteochondromatosis. J Pediatr Ortho.

[2] Peterson HA (1994). Deformities and problems of the forearm in children with multiple hereditary osteochondromata. J PaediatrOrthop.

[3] Masada K, Tsuyuguchi Y, Kawai H, Kawabata H, Noguchi K, Ono K (1989). Operations for forearm deformity caused by multiple osteochondromas. J Bone Joint Surg (Br).

[4] Wood VE, Sauser D, Mudge D (1985). The treatment of hereditary multiple exostosis of the upper extremity. J Bone Joint Surg Am.

[5] Chin KR, Kharrazi FD, Miller BS, Chin KR, Kharrazi FD, Miller BS (2000). Osteochondromas of the distal aspect of the tibia or fibula. Natural history and treatment. J Bone Joint Surg Am.

[6] Milgram JW (1983). The origins of osteochondromas and enchondromas. A histopathologic study. ClinOrthop.

[7] Kivioja A, Ervasti H, Kinnunen J, Kaitila I, Wolf M, Böhling T (2000). Chondrosarcoma in a family with multiple hereditary exostoses. J Bone Joint Surg (Br).

[8] Bock GW, Reed MH (1991). Forearm deformities in multiple cartilaginous exostoses. Skeletal Radiol.

[9] Baek GH, Rhee SH, Chung MS, Lee YH, Gong HS, Kang ES (2010). Solitary intra-articular osteochondroma of the finger. J Bone Joint Surg Am.

[10] Klekamp J, Green NE, Mencio GA (1998). Osteochondritisdissecans as a cause of developmental dislocation of the radial head. Clin Orthop Relat Res.

[11] Dotzis A, Galissier B, Peyrou P, Longis B, Moulies D (2009). Osteochondritisdissecans of the radial head: a case report. J Shoulder Elbow Surg.

[12] Tatebe M, Hirata H, Shinohara T, Yamamoto M, Morita A, Horii E (2012). Pathomechanical significance of radial head subluxation in the onset of osteochondritis dissecans of the radial head. J Orthop Trauma.

[13] Pierz KA, Stieber JR, Kasumi K, Dormans JP (2002). Hereditary multiple exotoses: one center’s experience and review of etiology. Clin Orthop Relat Res.

[14] Kim HT, Conjares JN, Suh JT, Yoo CI (2002). Chronic radial head dislocation in children, part 1: Pathologic changes preventing stable reduction and surgical correction. J Pediatr Ortho.

[15] Hirayama T, Takemisu Y, Yagihara K, Mikita A (1987). Operation for chronic dislocation of the radial head in children: reduction by osteotomy of the ulna. J Bone Joint Surg (Br).

[16] Seel MJ, Peterson HA (1999). Management of chronic post-traumatic radial head dislocation in children. J Pediatr Orthop.

[17] Hui JH, Sulaiman AR, Lee HC, Lam KS, Lee EH (2005). Open reduction and annular ligament reconstruction with fascia of the forearm in chronic Monteggia lesions in children. J Pediatr Orthop.

[18] Ladermann A, Cerioni D, Lefevre Y, De Rosa V, De Coulon G, Kaelin A (2007). Surgical treatment of missed Monteggia lesions in children. J Child Orthop.

[19] von Laer L, Halser C, Hell-Vocke KA (2005). Late missed Monteggia lesions: Reconstruction of the humeroradial joint. Eur J Trauma.

[20] Bhasker A (2009). Missed Monteggia fracture in children: Is annular ligament Reconstruction always required?. Indian J Orthop.

[21] De Boeck H (2000). Treatment of chronic isolated radial head dislocation in children. Clin Orthop Relat Res.

[22] Nakamura K, Hirachi K, Uchiyama S, Takahara M, Minami A, Imaeda T (2009). Long-term clinical and radiographic outcomes after open reduction for missed Monteggia fracture-dislocations in children. J Bone Joint Surg Am.

[23] Gyr BM, Stevens PM, Smith JT (2004). Chronic Monteggia fractures in children: outcome after treatment with the Bell-Tawse procedure. J Pediatr Orthop B.

[24] Eygendaal D, Hillen RJ (2007). Open reduction and corrective ulnar osteotomy for missed radial head dislocations in children. Strategies Trauma Limb Reconstr.

[25] Wang MN, Chang WN (2006). Chronic posttraumatic anterior dislocation of the radial head in children: thirteen cases treated by open reduction, ulnar osteotomy, and annular ligament reconstruction through a Boyd incision. J Orthop Trauma.

